# Complete Genomic Sequence of an Agarolytic *Pseudoalteromonas* Species Isolated from Deep Seawater

**DOI:** 10.1128/mra.00278-23

**Published:** 2023-06-21

**Authors:** Mikihisa Muta, Takao Yoshida, Takashi Funatsu, Ryo Iizuka

**Affiliations:** a Graduate School of Pharmaceutical Sciences, The University of Tokyo, Tokyo, Japan; b Japan Agency for Marine-Earth Science and Technology, Kanagawa, Japan; c Department of Biological Sciences, Graduate School of Science, The University of Tokyo, Tokyo, Japan; Montana State University

## Abstract

We report the complete genomic sequence of the agarolytic bacterium *Pseudoalteromonas* sp. strain MM1, recovered from deep seawater. The genome has two circular chromosomes with sizes of 3,686,652 bp and 802,570 bp and GC contents of 40.8 and 40.0%, and it carries 3,967 protein-coding sequences, 24 rRNA genes, and 103 tRNA genes.

## ANNOUNCEMENT

The genus *Pseudoalteromonas* comprises a group of marine bacteria that efficiently metabolize dispersed organic matter into growth substrates (1, 2). Here, we report the complete genomic sequence of *Pseudoalteromonas* sp. strain MM1, an agarolytic bacterium isolated from deep seawater.

Seawater was collected at a depth of 907 m off Hatsushima Island, Sagami Bay, Japan (35°0.940′N, 139°13.384′E), using the remotely operated vehicle (ROV) *Hyper-Dolphin* during cruise no. KM16-04 of the research vessel (R/V) *Kaimei* on 30 June 2016. The seawater was concentrated using centrifugal ultrafiltration (3), supplemented with 6% betaine, and stored at −80°C (4). The frozen stock was incubated in artificial seawater (GEX, Osaka, Japan) containing 0.05% yeast extract, 0.01% NH_4_Cl, and 1.5% ultralow gelling temperature agarose (Sigma-Aldrich) for 2 days at 30°C. The aliquot was streaked onto agar plates containing artificial seawater medium (5) and incubated for 4 days at 30°C, leading to the isolation of strain MM1, which exhibited a clear halo on the plates. Colony PCR was performed using the primers 27F (5′-AGAGTTTGATCMTGGCTCAG-3′) and 1492R (5′-TACGGYTACCTTGTTACGACTT-3′) to amplify the 16S rRNA gene. The sequence obtained (see “Data availability”) was identical to that found in Pseudoalteromonas tetraodonis GFC^T^ (GenBank accession no. CP011041). Genomic DNA was prepared using phenol-chloroform extraction from an overnight culture in artificial seawater medium at 20°C, followed by short- and long-read sequencing. For short-read sequencing, a library was constructed using the MGIEasy FS DNA library prep set (MGI). Paired-end sequencing (2 × 150 bp) was performed using the DNBSEQ-G400 high-throughput sequencing set (MGI) on a DNBSEQ-G400RS instrument (MGI). The raw data were filtered using fastp v.0.20.1 (6) (-q 30 -n 20 -t 1 -T 1). For long-read sequencing, genomic DNA (1 μg) was treated using the short-read eliminator kit XS (Circulomics) to remove fragments of <10 kbp, and a library was prepared using the ligation sequencing kit (Oxford Nanopore Technologies [ONT]). Sequencing was performed on a GridION X5 system using a FLO-MIN106 R9.4.1 flow cell (ONT). Base calling was conducted using Guppy v.6.4.6 (ONT). The raw data were processed using Filtlong v.0.2.1 (https://github.com/rrwick/Filtlong) (–min_length 3000 –keep_percent 90 –target_bases 500000000 –trim –split 500), specifying the option to use MGI reads. The resultant short- and long-read data were assembled using Unicycler v.0.4.8 (7), and the assembled sequences were polished using Pilon v.1.24 (8). Assembly errors were corrected manually using sequencing data. Automatic annotation was performed using DFAST v.1.2.18 (9, 10). All software was used with default parameters unless otherwise specified.

The MM1 genome comprised two circular chromosomes of 3,686,652 bp and 802,570 bp, harboring 3,967 protein-coding sequences, 24 rRNA genes, and 103 tRNA genes (Table 1). Whole-genome-based taxonomic analysis using autoMLST (11) suggested that MM1 represents a newly identified species within the genus *Pseudoalteromonas*, with a pairwise average nucleotide identity of 90.5% compared to the closest relative, Pseudoalteromonas espejiana DSM 9414^T^ (GenBank accession no. GCF_002221525). According to the results of dbCAN3 (12) analysis, the genome encodes various carbohydrate-active enzymes, including extracellular β-agarases (locus tags 18020, 18200, 18320, 18500, and 18510), which are likely involved in agar degradation.

**TABLE 1:** Genomic characteristics of the sequenced isolate

Type of results	Characteristic	Value
	BioSample accession no.	SAMD00589592
Short-read sequencing	No. of reads	
	Sequencing run 1	15,728,173
	Sequencing run 2	15,728,173
	Total length (bp)	
	Sequencing run 1	2,359,225,950
	Sequencing run 2	2,359,225,950
	SRA accession no.	DRR454389
Long-read sequencing	No. of reads	463,410
	Total length (bp)	1,941,613,951
	SRA accession no.	DRR454390
Assembly	Length (bp)	
	Chromosome 1	3,686,652
	Chromosome 2	802,570
	GC content (%)	
	Chromosome 1	40.8
	Chromosome 2	40.0
	Avg read depth (×)	
	Chromosome 1	319.0
	Chromosome 2	297.5
	No. of protein-coding sequences	
	Chromosome 1	3,238
	Chromosome 2	726
	No. of rRNAs	
	Chromosome 1	24
	Chromosome 2	0
	No. of tRNAs	
	Chromosome 1	103
	Chromosome 2	0
	GenBank accession no.	
	Chromosome 1	AP027922
	Chromosome 2	AP027923

### Data availability.

MM1 is associated with BioProject accession no. PRJDB15564, and the accession numbers are listed in Table 1. The 16S rRNA gene sequence can be found under GenBank accession no. LC767762.
